# Radical-SAM dependent nucleotide dehydratase (SAND), rectification of the names of an ancient iron-sulfur enzyme using NC-IUBMB recommendations

**DOI:** 10.3389/fmolb.2022.1032220

**Published:** 2022-10-21

**Authors:** Yuxuan Ji, Li Wei, Anqi Da, Holger Stark, Peter-Leon Hagedoorn, Simone Ciofi-Baffoni, Sally A. Cowley, Ricardo O. Louro, Smilja Todorovic, Maria Andrea Mroginski, Yvain Nicolet, Maxie M. Roessler, Nick E. Le Brun, Mario Piccioli, William S. James, Wilfred R. Hagen, Kourosh H. Ebrahimi

**Affiliations:** ^1^ Institute of Pharmaceutical Science, King’s College London, London, United Kingdom; ^2^ Institute for Pharmaceutical and Medicinal Chemistry, Heinrich-Heine-University Düsseldorf, Duesseldorf, Germany; ^3^ Department of Biotechnology, Delft University of Technology, Delft, Netherlands; ^4^ Magnetic Resonance Center (CERM), University of Florence and Consorzio Interuniversitario Risonanze Magnetiche di Metalloproteine (CIRMMP), Florence, and Department of Chemistry, University of Florence, Florence, Italy; ^5^ Sir William Dunn School of Pathology, University of Oxford, Oxford, United Kingdom; ^6^ Instituto de Tecnologia Química e Biológica António Xavier, Universidade Nova de Lisboa, Av. da República–EAN, Oeiras, Portugal; ^7^ Institute of Chemistry, Technische Universität Berlin, Berlin, Germany; ^8^ Université Grenoble Alpes, Grenoble, France; ^9^ Department of Chemistry, Molecular Sciences Research Hub, Imperial College London, London, United Kingdom; ^10^ Centre for Molecular and Structural Biochemistry, School of Chemistry, University of East Anglia, Norwich, United Kingdom

**Keywords:** antiviral, innate immun system, dehydratase, nucleotide analogue, iron-sulfur [FeS] cluster

## Main text

In 1789, the influential French chemist Antoine-Laurent Lavoisier described his view of science and its langague in his book *Traité élémentaire de chimie*. According to the Robert Kerr’s translation it states (Lavoisier, 1790): “*As ideas are preserved and communicated by means of words, it necessarily follows that we cannot improve the language of any science without at the same time improving the science itself; neither can we, on the other hand, improve a science without improving the language or nomenclature which belongs to it*.” This view reminds us of Confucius’s earlier doctrine, the rectification of names (Steinkraus, 1980; Lau, 2000). Confucius believed that rectification of names is imperative. He explained (Steinkraus, 1980; Lau, 2000): “*If language is incorrect, then what is said does not concord with what was meant, what is to be done cannot be affected. If what is to be done cannot be affected, then rites and music will not flourish. If rites and music do not flourish, then mutilations and lesser punishments will go astray. And if mutilations and lesser punishments go astray, then the people have nowhere to put hand or foot. Therefore the gentleman uses only such language as is proper for speech, and only speaks of what it would be proper to carry into effect. The gentleman in what he says leaves nothing to mere chance.*” Inspired by these views, we make the analogy that the progress of science and the language used to describe it are two entangled electrons. This entanglement highlights the importance of introducing systemic names for enzymes using EC classification and the ever-growing problem of protein names (McDonald and Tipton, 2021). Here, we tackle one specific case of iron-sulfur ([FeS]) enzymes. We show that the language used to describe a conserved [FeS] enzyme of the innate immune system, i.e., viperin or RSAD2, is now inadequate and disentangled from its science. We discuss that the enzyme has cellular functions beyond its antiviral activity and that eukaryotic and prokaryotic enzymes catalyse the same chemical reactions. To prevent bias towards antiviral activity while studying various biochemical activities of the enzyme and using scientifically incorrect terms like “prokaryotic viperins,” we rectify the language describing the enzyme. Based on NC-IUBMB recommendations, we introduce the nomenclature S-adenosylmethionine (SAM) dependent Nucleotide Dehydratase (SAND).

**FIGURE 1:**
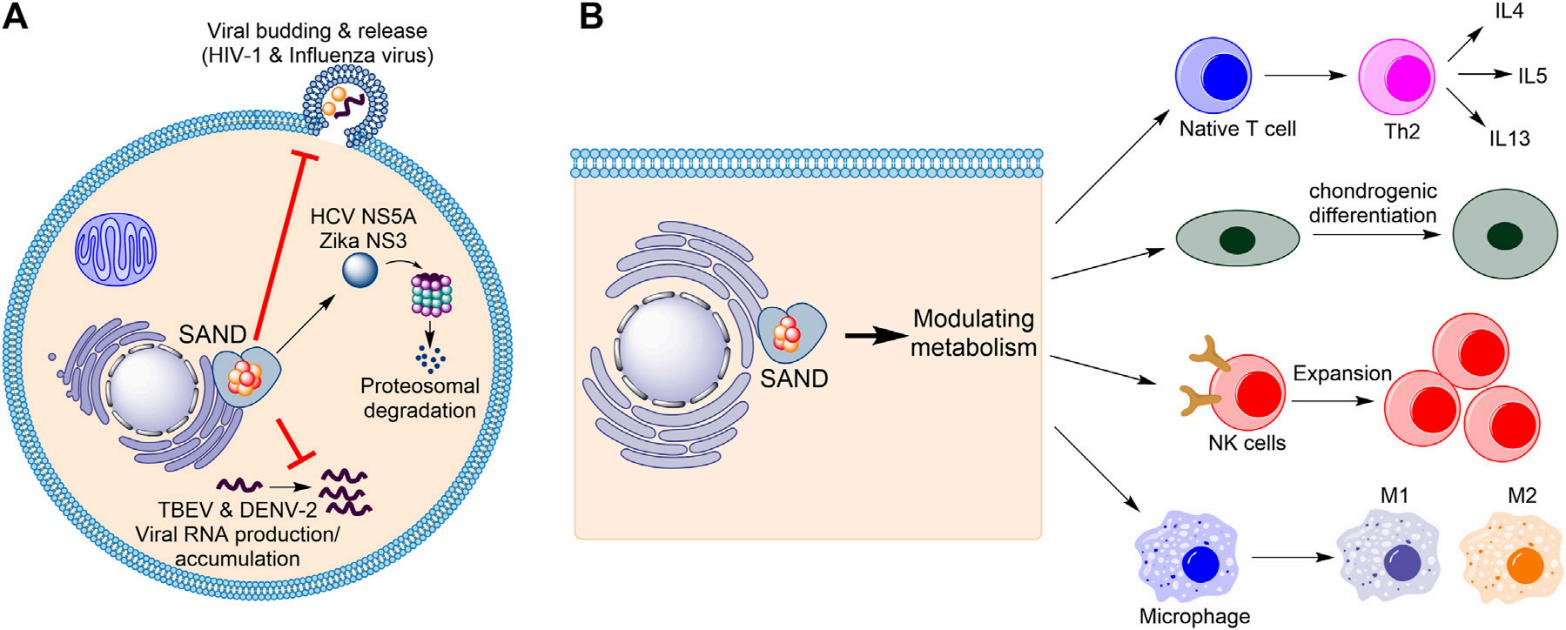
Human SAND has functions beyond its antiviral activity. **(A)** Various mechanisms of antiviral activity are proposed for human SAND. **(B)** Expression of SAND affects the function and differentiation of various types of cells. HCV, hepatitis C virus; TBEV, tick-born encephalitis virus; DENV-2, Dengue type-2 virus; NK, natural killer; IL, interleukin.

Firstly, considering the progress in understanding the biology of the enzyme in humans (Figure 1), the name “viperin” is no longer adequate and should be avoided. In 1997, Hua Z., et al. found that in response to human cytomegalovirus infection, the mRNA level of a novel protein was elevated in human cells (Zhu et al., 1997). The gene related to this mRNA was named cytomegalovirus-induced human gene-5 (cig-5). In 2001, Chin and Cresswell showed that interferons (IFNs) induce the expression of the protein product of cig-5 (Chin and Cresswell, 2001). This induction restricted the replication of human cytomegalovirus, and the protein was localised to the cytoplasmic face of the endoplasmic reticulum (ER) (Chin and Cresswell, 2001). Because, at the time, nothing was known about the chemistry of the enzyme, an abbreviation based on the cellular localisation and antiviral activity was introduced, “viperin” (virus inhibitory protein, endoplasmic reticulum-associated, interferon-inducible) (Chin and Cresswell, 2001). Subsequent studies showed that the expression of the protein affects the life-cycle of many RNA and DNA viruses, including Influenza (Wang et al., 2007), HIV-1 (Nasr et al., 2012), Hepatitis C (Wang et al., 2012; Ghosh et al., 2020), Zika (Van der Hoek et al., 2017; Panayiotou et al., 2018), and tick-borne encephalitis (Panayiotou et al., 2018), among others. However, for each virus, different mechanisms were proposed (Figure 1A). For example, the enzyme affects lipid rafts (lipid microdomains on the cellular membrane and enriched in cholesterol and sphingolipids (Ripa et al., 2021)) and inhibits influenza virus (Wang et al., 2007) or HIV-1 (Nasr et al., 2012) release. In the case of the Hepatitis C virus, viperin expression appears to interfere with the binding of the viral nonstructural protein NS5A with host hVAP22 (Wang et al., 2012) or promotes proteasomal-dependent degradation of viral NS5A (Ghosh et al., 2020). In the case of Zika and tick-borne encephalitis viruses, the enzyme appears to induce proteasomal degradation of the viral nonstructural protein NS3 (Panayiotou et al., 2018). Finally, in the case of tick-borne encephalitis virus and Dengue virus type-2, the enzyme restricts viral RNA reproduction (Helbig et al., 2013; Upadhyay et al., 2014).

Despite the established antiviral activity, many studies have reported biological functions inconsistent with or unrelated to the biology defined by the nomenclature “viperin”. Cresswell and colleagues showed that the enzyme localises to lipid droplets (Hinson and Cresswell, 2009) and enhances human cytomegalovirus infection (Seo et al., 2011). In addition to interferons, lipopolysaccharides were found to induce protein expression (Olofsson et al., 2005). The proposal of multiple mechanisms of antiviral activity prompted us to postulate that the protein’s enzymatic activity regulates metabolism to affect various cellular processes causing broad-spectrum antiviral activity (Ebrahimi, 2018) (Figure 1B). This effect of the enzyme on metabolism suggests a cellular function beyond its antiviral activity. Indeed, many studies corroborate this proposal and demonstrate that human SAND has a role in modulating metabolism, regulating the activity/maturation of the immune cells, and inducing the expression of immune genes (Figure 1B). For example, the activity of SAND modulates central carbon metabolism (Ebrahimi et al., 2020c), regulates thermogenesis in adipose tissues (Eom et al., 2019), inhibits thiolase activity of the trifunctional enzyme complex (a mitochondrial enzyme complex with three activities: enoyl-CoA hydratase, 3-hydroxyacyl-CoA dehydrogenase, and 3-ketoacyl-CoA thiolase) (Dumbrepatil et al., 2020), and modulates cholesterol metabolism (Tang et al., 2016; Grunkemeyer et al., 2021). It is required for optimal T helper two cell response (Qiu et al., 2009) and chondrogenic differentiation *via* CXCL10 protein secretion (Steinbusch et al., 2019). It has a role in the innate system (Ebrahimi et al., 2022) and modules immune cell function and maturation e.g., expansion of natural killer cells (Wiedemann et al., 2020), dendritic cell maturation (Jang et al., 2018), B cell hyperactivity (Zhu et al., 2021), and polarisation of macrophages (Eom et al., 2018). Additionally, the enzyme’s expression induces the expression of many immune genes (Zhang et al., 2014).

**FIGURE 2:**
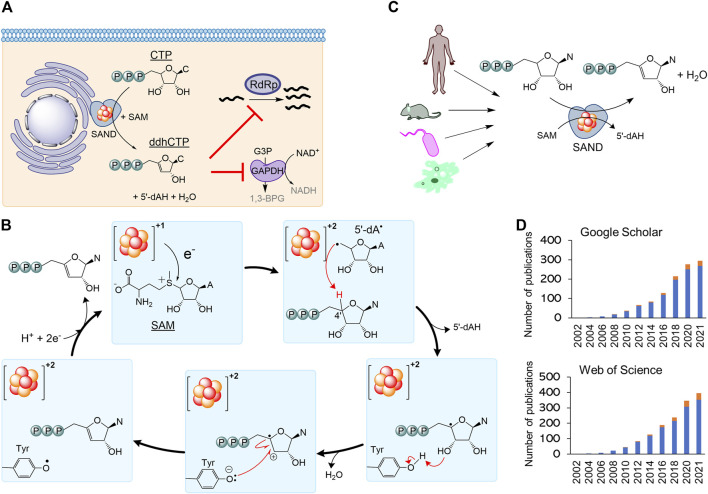
The nomenclature SAND (SAM-dependent Nucleotide Dehydratase) defines chemistry relevant to biology across all domains of life. **(A)** SAND produces the nucleoside triphosphate analogue ddhCTP in humans. ddhCTP modulates metabolism affecting cell function and restricting viral replication. **(B)** The proposed mechanism of dehydration of nucleoside triphosphates by SAND. The mechanism shows the transfer of a proton and an electron from a conserved tyrosine. Alternatively, it is possible that proton transfer occurs *via* another amino acid residue. It is not clear if the transfer of proton and electron occurs simultaneously (proton-coupled electron transfer). **(C)** SANDs from various organisms produce diverse ddh analogues. **(D)** An increasing number of investigators study SANDs. The data were obtained from a search of nomenclature viperin and RSAD2 in the title of articles. Google Scholar (scholar.google.com) and Web of Science search engines were used. N, nucleobase; C, cytosine; A, adenine; 5′-dAH, 5′-deoxyadenosine, 5′-dA·, 5′-deoxyadenosyl radical.

Secondly, the nomenclature RSAD2 should be revised to fully describe the chemistry of the enzyme relevant to its biological function. By 2010, it became clear that human SAND has a CxxxCxxC motif coordinating a [4Fe-4S] cluster, similar to many members of the radical S-adenosylmethionine (SAM) enzymes (Duschene and Broderick, 2010; Shaveta et al., 2010). As a result, the HUGO Gene Nomenclature Committee suggested the name RSAD2 (radical-SAM domain containing 2) around this time. This name can be easily confused with another radical-SAM enzyme of unknown function (RSAD1), and it only partially describes the SAM-dependent chemistry of the enzyme. In 2017, the structure of mouse SAND was solved (Fenwick et al., 2017), confirming that it is a radical-SAM enzyme. It was shown that the cytosolic iron-sulfur biogenesis machinery is required to deliver and insert the [4Fe-4S] cluster into the enzyme (Upadhyay et al., 2017). The expression of human SAND in *E. coli* changed the cells’ morphology, suggesting the enzyme’s substrate is a metabolite common between eukaryotic and prokaryotic cells (Nelp et al., 2017)*,* and initial structural studies proposed that the substrate is a nucleotide (Fenwick et al., 2017). Subsequently, it was revealed that eukaryotic SAND could catalyse the dehydration of CTP or UTP to 3ʹ-deoxy-3ʹ, 4ʹ-didehydro (ddh) analogues (Figure 2A) (Fenwick et al., 2020). In human macrophages, the enzyme was found to produce ddhCTP (Gizzi et al., 2018; Ebrahimi et al., 2020b). This novel nucleotide analogue metabolite may act as a chain-terminator to inhibit viral replication (IC_50_ values ≥ 20 mM) (Gizzi et al., 2018). Subsequent studies revealed that the expression of SAND and synthesis of ddhCTP in HEK293 cells affects the cellular nucleotide pool and mitochondrial function (Ebrahimi et al., 2020a). The enzyme in macrophages modulates central carbon metabolism potentially by inhibiting the NAD^+^-dependent activity of the glycolytic enzyme GAPDH (Ebrahimi et al., 2020c) (Figure 2A). This function requires the radical-SAM domain to produce ddhCTP since this nucleotide analogue inhibits the NAD^+^-dependent activity of GAPDH *in vitro* (Ebrahimi et al., 2020c). This immunometabolism function of ddhCTP may regulate the immune response in various ways (Ebrahimi et al., 2021, Ebrahimi et al., 2022). Consistently, studies have shown that the expression of the enzyme indeed primes the immune response (Zhang et al., 2014).

Finally, the use of the outdated nomenclature “viperin” can introduce scientifically incorrect terms such as “prokaryotic viperin.” Before 2017 little was done to isolate fungal and microbial SANDs and characterise the chemical reaction catalysed by them. In 2017, a thermostable fungal SAND from *Thielavia terrestris* was isolated and characterised (Ebrahimi et al., 2017). It was hypothesised that the fungal enzyme produces antiviral natural products and is a suitable candidate for the biotechnological production of antiviral lead molecules. The fungal SAND has promiscuous activity and catalyses the dehydration of diverse nucleoside triphosphates (NTPs), e.g., CTP, UTP, and 5-bromo-UTP, to their ddh analogues *via* a mechanism requiring the transfer of an electron and a proton (Figure 2B) (Ebrahimi et al., 2020b). Next, a number of other groups characterised some microbial enzymes and showed that they catalyse dehydration of various NTPs to their ddh analogues (Bernheim et al., 2021; Lachowicz et al., 2021) (Figure 2C). While the cellular function of these microbial proteins is not fully understood, the chemical reaction catalysed by SANDs can inhibit the activity of phage T7 RNA polymerase in *E. coli* (Bernheim et al., 2021). These findings suggest that the enzyme might have a cellular function and act as an antimicrobial/antiviral defence system. The fungal enzyme was named TtRSAD2 (Ebrahimi et al., 2020b) due to the lack of a proper name, and studies with bacterial enzymes (Bernheim et al., 2021) introduced a new nomenclature, i.e., “prokaryotic viperin,” to describe prokaryotic enzymes producing ddh analogues with antiviral activity (Bernheim et al., 2021; Wein and Sorek, 2022). The term “prokaryotic viperin” is not fit for purpose because it implies that bacteria and archaea have endoplasmic reticulum, and interferons activate their immune system. This assertion questions our fundamental understanding of biology, i.e., prokaryotes do not have an endoplasmic reticulum and interferon-mediated antiviral response.

A growing number of investigators are studying this new class of enzymes across all domains of life (Figure 2D). Consequently, different nomenclatures like RSAD2, viperin, prokaryotic viperin, or viperin-like enzymes are being used by various investigators, including us, to describe eukaryotic or microbial enzymes. As discussed above, none of the existing nomenclatures accurately describe the cellular function or chemistry in prokaryotes or eukaryotes. Additionally, using various terminologies for enzymes performing the same chemical reaction is confusing. Hence, we strongly suggest the classification of the enzyme as a nucleoside triphosphate dehydratase (NTPD, EC 4.2.1) and the nomenclature SAND describing the SAM-dependent chemistry across all domains of life. This classification and abbreviation to rectify the naming of an ancient iron-sulfur enzyme should help the increasing number of investigators studying the cellular function or biotechnological application of these enzymes and the discovery of new enzymes performing novel chemistries.
