# Supplementary figures and images for: Radical-SAM dependent nucleotide dehydratase (SAND), rectification of the names of an ancient iron-sulfur enzyme using NC-IUBMB recommendations
Source: Front Mol Biosci. 2022 Oct 21;9:1032220. doi: 10.3389/fmolb.2022.1032220 (PMC9642334; doi:10.3389/fmolb.2022.1032220)

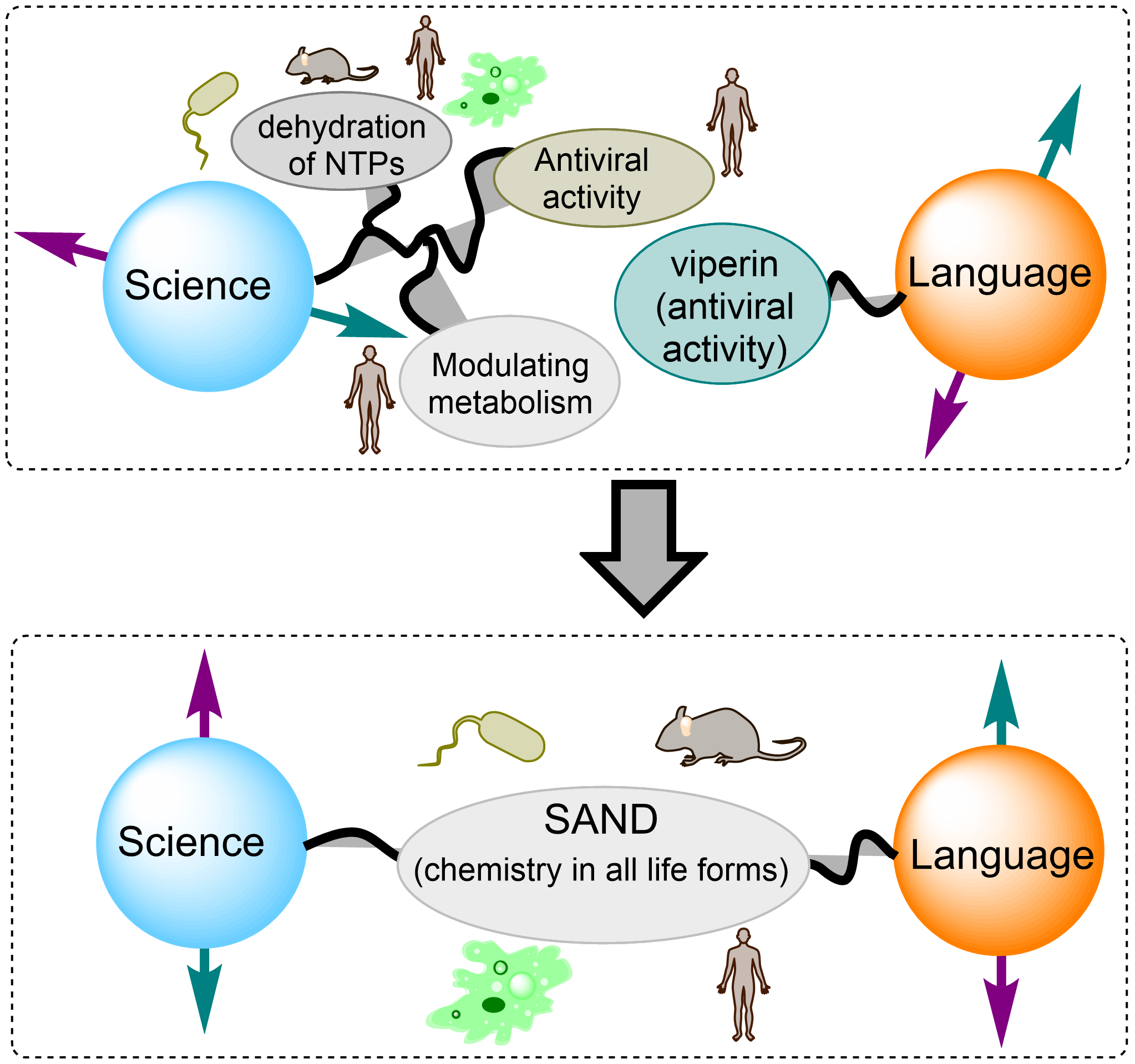

Supplement: Supplementary file 1 [file Image1.TIF]
